# Dental management before radiotherapy of the head and neck region: 4‐year single‐center experience

**DOI:** 10.1002/cre2.662

**Published:** 2022-09-11

**Authors:** Lea Hoffmann, Sebastian N. Marschner, Tamara K. Kakoschke, Reinhard Hickel, Hisham Sabbagh, Uta C. Wölfle

**Affiliations:** ^1^ Department of Conservative Dentistry and Periodontology, University Hospital LMU Munich Munich Germany; ^2^ Department of Orthodontics and Dentofacial Orthopedics, University Hospital LMU Munich Munich Germany; ^3^ Department of Radiation Oncology, University Hospital LMU Munich Munich Germany; ^4^ German Cancer Consortium (DKTK) Partner Site Munich Munich Germany; ^5^ Department of Oral and Maxillofacial Surgery and Facial Plastic Surgery, University Hospital LMU Munich Munich Germany

**Keywords:** dental management, follow‐up, oral cancer, radiotherapy

## Abstract

**Objective:**

To review our experience with a standardized dental management approach in patients with planned radiotherapy of the head and neck region based on preradiation and follow‐up data.

**Material and Methods:**

Records of patients who underwent radiotherapy between June 2016 and November 2020 were reviewed. Data on dental findings and therapeutic recommendations were extracted from a prospectively managed database. Hospital records were used to obtain follow‐up data.

**Results:**

Two hundred eighty‐one patient records were identified. After the exclusion of 81 patients because of incomplete data, 200 patients were included in the study. Dental findings relevant to radiotherapy were found in 144 cases (72.0%). Teeth extractions were recommended in 112 (56.0%) patients. Follow‐up data were available for 172 (86.0%) patients (mean follow‐up: 16.8 ± 10.7 months). Radiodermatitis was the most frequently observed sequela of radiotherapy (42.4%), followed by dysphagia (38.4%) and stomatitis (36.6%). Osteoradionecrosis was observed in only 2.3% of the patients.

**Conclusion:**

Dental findings relevant to planned radiotherapy were frequent and in many cases resulted in recommendations for teeth extraction. Based on our standardized dental management protocol, we observed low rates of late oral complications after radiotherapy of the head and neck region.

## 1 INTRODUCTION

Radiotherapy is a standard form of treatment for many head and neck tumors. Depending on the tumor entity, size, stage, localization, grade, resection status, and patient comorbidities, radiotherapy can be applied as an adjuvant or alternative to surgery. Where indicated, it can be combined with chemotherapy (Koga et al., 2008). Radiotherapy leads to a decreased proliferation rate or even apoptosis: the tumor stops growing or decays. Nevertheless, the cell‐damaging effects of radiation therapy are nonspecific, and the tumor‐surrounding tissue (i.e., oral mucosa, salivary glands, maxillary and mandibular bone, teeth, and masticatory muscles) is affected as well (Eriksson & Stigbrand, 2010). These impairments are dependent on the radiation dose (single or cumulative), duration of treatment, and individual sensitivity of the tissue (Brook, 2020). The recommended total dose of curative radiation treatment in head and neck cancers is 60–80 Gray (Gy). Dose fractionation (wherein the total radiation dose is not applied in one session but is divided into a number of smaller doses administered over a defined period of time) reduces negative side effects on healthy tissue while maintaining the maximum impact on cancer cells. Using conventional fractionation, the total dose of approximately 70 Gy is divided into daily single fractions of 1.8–2 Gy applied five times per week (Bourhis et al., 2005; Dionisi et al., 2019).

Side effects caused by radiotherapy can be divided into acute and chronic damage (Wang & Tepper, 2021). Some of the side effects of radiotherapy on the head and neck region are unavoidable. However, factors such as caries or osteoradionecrosis of the jaw (ORNJ) may potentially be prevented by accurate dental management before irradiation (Beech et al., 2014; Katsura et al., 2008). Thus, it is recommended that teeth with carious lesions, especially deep defects that extend to the pulp (category D4) (Manton, 2013), with a risk for pulpitis and developing ORNJ, be treated or removed before radiotherapy (Beech et al., 2014; Gehrig, 1969; Kielbassa et al., 2006). Furthermore, periodontal disease should be managed, including extractions of teeth with periodontal pockets of ≥6 mm (Beech et al., 2014; Katsura et al., 2008), or endodontic treatments of teeth with apical osteitis should be carried out (Grötz, 2002). For a prompt start of irradiation, extraction must be considered as a rapid therapeutic alternative to endodontic treatment or other tooth restorations with poor prognosis (Beech et al., 2014). Apart from the recommendations mentioned above, there are no consistent guidelines for dental management before radiotherapy. The aim of this study is to review our single‐center experiences with a standardized approach for dental management in patients before radiotherapy in the head and neck region as well as to review the frequency of side effects after radiotherapy in patients available for follow‐up.

## 2 METHODS

This retrospective study was approved by the ethics committee of Ludwig Maximilian University (approval number 20‐778). Between June 2016 and November 2020, data from patients who underwent dental screening before radiotherapy of the head and neck region in the Department of Conservative Dentistry and Periodontology of University Hospital were extracted. For dental screening, a standardized protocol is used at the Department of Conservative Dentistry and Periodontology. The dental examination included documentation of decayed/missing/filled teeth (DMFT) status, 6‐point pocket measurement, percussion test, and pulp sensitivity test. Furthermore, clinical and radiological findings (fillings, crowns, partial crowns, bridge elements, replaced teeth, missing teeth, carious lesions, destroyed teeth, implants, periodontal pockets >6 mm, radiological indications for root canal treatment [adequate/inadequate], and radiological indications for osteolysis), as well as the recommended therapy, were documented.

The screening protocol and recommended treatment were based on recommendations published by Ben‐David et al. (2007), who stated that “teeth with nonrestorable caries or caries that extend to the gum line, teeth with large, compromised restorations with significant periodontal attachment loss (pocketing >5 mm) and those with severe erosion or abrasion should be extracted if they are in parts of the jaws expected to receive a high dose of radiation.” Slightly deviating from these recommendations, we considered periodontal pockets deeper than 6 mm to be critical. An orthopantomogram was performed to assess the radiological findings of teeth and bone condition. If indicated, further small X‐rays of single teeth and the surrounding area (intraoral radiographic views) were taken. The mucosa, gingiva, and excretory ducts of salivary glands were visually examined. Screening results were controlled by the senior physician on duty. Table 1 summarizes the criteria for dental therapy recommendations.

**Table 1:** Findings and dental therapeutic recommendations

Finding	Therapeutic recommendation
Carious with no extension to the pulp	New filling/Extraction when opening the pulp
Carious with extension to the pulp	Extraction
Insufficient crowns, bridges, or fillings	Repair or renewal
Periodontal pockets >4 mm and <6 mm	Periodontitis therapy
Periodontal pockets >6 mm	Extraction
Adequate root canal treatment	No therapy
Inadequate root canal treatment	Root apex resection/extraction
Apical osteolysis of a tooth	Root canal treatment/extraction
Periimplantitis	Explanation
Unclear findings of the bone	Coevaluation by the Department of Maxillofacial Surgery
Insufficient denture (sharp edges)	New denture

Cancer diagnosis, radiation dose, and field were provided by the Department of Radiation Oncology. Intensity‐modulated radiotherapy (IMRT) or volumetric‐modulated arc therapy (VMAT) techniques were used for patient irradiation.

Data were extracted from the database mentioned above (L. H., U. C. W.) and retrospectively analyzed using Excel software (Microsoft, Redmond, USA). Data anonymization and data processing were performed by a program written specifically for this purpose (Python Software, Version 3, Delaware, USA). Only patients with a complete data set containing all the measurement parameters mentioned above were included in the study.

Follow‐up was performed by reviewing hospital records up to November 2020. Complications after irradiation attributable to radiotherapy (oral candidiasis, stomatitis, dysphagia, radiodermatitis, xerostomia, oral ulcers, osteoradionecrosis) were identified and added to the patient's data set. Complications were identified based on clinical observations and patient self‐reports as documented in the patient's record. As hospital records did not include a dental status and further dental treatment in most of our patients took place outside our clinic in an outpatient setting (private practice), we were not able to analyze whether or not our dental treatment recommendations were followed.

As this study was a retrospective study, the evaluation and severity of the follow‐up findings were based on the assessment of the investigators.

Descriptive statistics of the data were obtained with SPSS (Version 26, Chicago, USA). Pearson's *χ* ^2^ test was used to analyze significant differences between the dental findings, treatment recommendations, complications after radiotherapy, and gender or cancer type. A *p* value < .05 was considered to be significant.

## 3 RESULTS

### 3.1 Patient cohort

The database included 281 patients with planned radiotherapy of the head and neck region between June 2016 and November 2020. Eighty‐one patients were excluded from the analysis because of incomplete data. Table 2 shows the characteristics of the 200 patients with complete data included in the study. Seventy‐one percent were men, 29.0% were women, and the mean age was 61.3 ± 12.7 years. Most patients had squamous cell carcinoma (59.0%), followed by salivary gland carcinoma (8.0%) and lymphoma (6.5%), and the average planned irradiation dose was 61.3 ± 12.6 Gy. The radiation field affected the salivary glands (parotid, submandibular, and sublingual) completely or partly. Dental clinical examination yielded pathological findings regarding the lining mucosa in 25 patients (12.5%) due to previous tumor surgery, such as swelling, stitches, persisting wounds after surgery (9.5%), and tumor manifestations (2%). Salivary glands were associated with only a few pathological findings (1.5%–2.5%), which were mainly due to previous surgery in this area. Tables 3 and 4 present the dental findings in detail. Table 5 summarizes the recommended dental therapies before radiotherapy. In total, pathological dental findings relevant to planned therapy were found in 144 cases (72.0%). Teeth extraction was recommended in 112 patients (56.0%), with an average of 4.43 teeth per patient. In nine patients (4.5%), filling therapy was suggested. If pulp opening due to a deep carious lesion occurred during planned filling therapy, extraction was recommended. All patients received a recommendation for professional dental cleaning, including ultrasonic cleaning, tartar removal, and teeth polishing.

**Table 2:** Patient characteristics; age (in years) and planned irradiation dose (in gray) are given as the mean± standard deviation

		N (%)
Patients included		200 (100)
Age (years)	65.6 ±13.1	
Sex	Female	58 (29.0)
	Male	142 (71.0)
Disease	Squamous cell carcinoma	118 (59.0)
	Salivary gland carcinoma	16 (8.0)
	Lymphoma	13 (6.5)
	CUP syndrome	9 (4.5)
	Metastasis	5 (2.5)
	Spinalioma	5 (2.5)
	Melanoma	3 (1.5)
	Neuroendocrine carcinoma	3 (1.5)
	Plasmacytoma	3 (1.5)
	Sarcoma	3 (1.5)
	Glomus jugulare carcinoma	3 (1.5)
	Other disease/no specific designation^a^	19 (9.5)
Metastasized^b^		24 (12.0)
Cancer recurrence^b^		18 (9.0)
Planned irradiation dose in Gray	61.3 ±12.7	

Abbreviation: CUP = carcinoma with unknown primary tumor.

^a^ Clivus meningioma (*n* = 1), multiple myeloma (*n* = 2), neuroblastoma (*n* = 1), osteomeningioma (*n* = 1), thyroid carcinoma (*n* = 1), synovial sarcoma (*n* = 1), no specific designation (*n* = 12).

^b^ At the time of the dental focus search, metastases or a recurrence was already present.

**Table 3:** Clinical findings

	*N* (%)
Mobile mucosa	
Without pathological findings	175 (87.5)
With pathological findings	25 (12.5)
• Change due to surgery^a^	19 (9.5)
• Manifestation of the tumor	4 (2)
• Morsicatio	2 (1)
Parotid gland	
Without pathological findings	197 (98.5)
With pathological findings	3 (1.5)
• Condition after surgery^a^	3 (1.5)
Submandibular gland	
Without pathological findings	196 (98.0)
With pathological findings	4 (2.0)
• Condition after surgery^a^	1 (0.5)
• No specific designation	3 (1.5)
Sublingual gland	
Without pathological findings	195 (97.5)
With pathological findings	5 (2.5)
• Condition after surgery^a^	1 (0.5)
• Slightly enlarged	1 (0.5)
• No specific designation	3 (1.5)
Right parotid gland within the radiation area	
Data not given	62 (31.0)
Completely	49 (24.5)
None	30 (15.0)
Partly	59 (29.5)
Left parotid gland within the radiation area	
Data not given	62 (31.0)
Completely	40 (20.0)
None	28 (14.0)
Partly	70 (35.0)
Right submandibular/sublingual gland within the radiation area	
Data not given	62 (31.0)
Completely	73 (36.5)
None	25 (12.5)
Partly	40 (20.0)
Left submandibular/sublingual gland within the radiation area	
Data not given	62 (31.0)
Completely	67 (33.5)
None	27 (13.5)
Partly	43 (21.5)

^a^ Swelling, stitches, or wounds with a clear conclusion of surgery.

**Table 4:** Dental findings before radiotherapy (mean± SD)

Finding	Mean ± SD
DMFT	20.07 ± 6.3
Existing fillings	5.11 ± 4.96
Existing partial crown	0.61 ± 1.76
Existing crown	3.84 ± 4.49
Existing bridge element	0.96 ± 1.90
Replaced teeth (prosthetic replacement)	0.70 ± 3.60
Missing teeth	11.80 ± 8.37
Carious lesions	2.03 ± 2.99
Destroyed teeth (that could not be restored)	0.34 ± 1.22
Implant	0.54 ± 1.56
Periodontal pocket ≥6 mm maxillary^a^	1.39 ± 3.62
Periodontal pocket ≥6 mm mandibular^a^	1.21 ± 3.48
Radiological indications of adequate root canal treatment	1.88 ± 1.38
Radiological indications of inadequate root canal treatment	1.59 ± 0.86
Radiological indications of (apical) osteolysis	1.50 ± 1.03

Abbreviation: DMFT, decay‐missing‐filled teeth index.

^a^ With a 6‐point pocket measurement carried out.

**Table 5:** Recommended dental therapy before radiotherapy

Recommended therapy	*N* a (%)
Findings clinically relevant before irradiation	
Yes	144 (72.0)
No	56 (28.0)
Extraction	112 (56.0)
Recommended extraction after unsuccessful treatment	9 (4.5)
Root canal treatment	10 (5.0)
Root apex resection	1 (0.5)
Explanation	3 (1.5)
Direct filling	56 (28.0)
Periodontitis therapy	53 (26.4)
Irradiation/fluoridation splint	91 (45.3)
Coevaluation by the Department of Maxillofacial Surgery due to unclear findings	14 (7.0)
New dentures	15 (7.5)

^a^ Counts were based on whether a therapy was indicated. For example, if a patient had several teeth to be extracted, the situation was evaluated as one necessary extraction therapy. The percentages refer to the *n* = 144 relevant cases.

### 3.2 Follow‐up

Follow‐up data were available for 172 patients. Twenty‐eight patients were lost to follow‐up (14.0%). The mean follow‐up was 16.8 ± 10.7 months (range 1–49 months). Nine patients (5.2%) died, and 21 patients (12.2%) had their last follow‐up visit more than a year ago. Table 6 summarizes the results of the follow‐up investigations. Radiodermatitis was the most frequently observed sequela of radiotherapy (73 patients; 42.4%), followed by dysphagia (66 patients; 38.4%) and stomatitis (63 patients; 36.6%). ORNJ was observed in only 2.3% of the patients.

**Table 6:** Follow‐up

Follow‐up	*N* (%)
Yes	172 (86.0)
No	28 (14.0)
Last check‐up <1 year	142 (82.6)
Last check‐up >1 year	21 (12.2)
Death	9 (5.2%)
New metastasis	18 (10.5)
New cancer recurrence	15 (8.7)
Oral candidiasis	36 (26.7)
Stomatitis	63 (36.6)
Dysphagia	66 (38.4)
Radiodermatitis	73 (42.4)
Xerostomia	50 (29.1)
Oral ulcers	8 (4.7)
Osteoradionecrosis	4 (2.3)

No significant differences were observed between the dental findings, treatment recommendations, complications after radiotherapy, and genders or cancer types.

## 4 DISCUSSION

Tumors in the head and neck area are one of the most common types of cancer worldwide (Jemal et al., 2011; Siegel et al., 2013). Alcohol, tobacco, and human papillomavirus are regarded as the main risk factors for the development of oral cancer (Ang et al., 2010; Hashibe et al., 2009). Squamous cell carcinoma is the most common type of oral tumor, accounting for 90% of all oral cancers (Montero & Patel, 2015). In western regions, men show a threefold higher risk of developing oral tumors than women; these tumors usually develop after the fifth decade of life (Kruse et al., 2011; Montero & Patel, 2015). However, in recent years, there has been an increase in the tumor rate in women, probably due to the increasing exposure of women to alcohol and tobacco (Conway et al., 2018).

These findings are in line with those of the patient cohort described in the present study. However, the prevalence of squamous cell carcinoma of 59% observed in our study was substantially lower than the 90% prevalence reported previously (Montero & Patel, 2015).

Oral hygiene indicators, such as missing teeth, denture use, bleeding gums, infrequent dental visits, and lack of tooth brushing, have an impact on the etiology of cancer in the head and neck region independent of alcohol and tobacco use (J. S. Chang et al., 2013; Hashim et al., 2016; Manoharan et al., 2014). The findings in the present cohort mirror these observations. Initial dental examination revealed a mean of 2.6 periodontal pockets > 6 mm per patient. Furthermore, the mean DMFT value was 20.07 ± 6.3. A lower DMFT value of 9.2 was reported in a systematic review of dental disease in patients undergoing cancer therapy (Hong et al., 2010). The higher DMFT value in our study is most likely due to the higher number of missing teeth, with an average of 11.80 ± 8.4 missing teeth per patient.

The aim of dental management in patients undergoing radiotherapy of the head and neck region is to create a satisfactory esthetic and functional situation. Timely planning of preventive measures and treatments is crucial, as the time interval between the decision to treat and the initiation of radiotherapy is often short. Irradiation results in a weakened immune system, reduced blood flow, and decreased saliva production. Thus, surgical wounds, extractions, apical and marginal periodontitis, denture pressure points, and ulcerations may lead to an increased risk of osteoradionecrosis (Bornstein et al., 2001).

Most patients with head and neck carcinomas treated with curative intent receive a dose of 2 Gy per fraction delivered five times per week, up to a total dose of 64–70 Gy (Bourhis et al., 2005). Although there are no guidelines on the dental management of patients with planned radiotherapy of the head and neck region, it is generally acknowledged that dental management should consider the region and radiation dose planned, as the severity of oral complications is related to the daily and total cumulative dose of radiation (Haussmann et al., 2019; Rapp et al., 2020; Sciubba & Goldenberg, 2006). Moreover, the prevention of dental complications should include oral hygiene instruction, scaling, cleaning, and intensive fluoridation. Restorations in need of repair should be treated before radiotherapy. Furthermore, sharp edges on teeth or dentures should be smoothed to avoid ulcerations (Beech et al., 2014).

In 144 out of our 200 patients, clinically relevant findings before irradiation were observed, which mostly resulted in a therapeutic recommendation for extraction (Table 5). Our findings are in line with previous studies where 58%–97% of patients required some kind of dental treatment before planned radiotherapy (Bertl et al., 2016; Critchlow et al., 2014; Jham et al., 2008). As in our study hospital records did not include a dental status and further dental treatment in most of our patients took place outside our clinic in an outpatient setting (private practice), we had no information to what extent our recommendations were followed.

There are only limited data at which rate required dental treatment recommendations are subsequently implemented. As Bertl et al. reported that about 90% of patients followed dental recommendations involving a multidisciplinary treatment team, we may assume that in most of our patients' recommendations before radiotherapy we mostly adequately followed (Bertl et al., 2022).

Extraction is considered to be indicated in cases of extensively destroyed teeth, whether due to caries, loss of periodontal structures, questionable pulpal state, residual roots, or incompletely erupted teeth with contact with the oral cavity (Ben‐David et al., 2007; Moore et al., 2020; Schuurhuis et al., 2015). However, there are inconsistent results among studies regarding extractions before radiotherapy and their association with the development of ORNJ. In some studies, extractions before radiotherapy have been reported to reduce the risk of ORNJ (Chopra et al., 2011), while in other studies, it has been concluded that extractions before radiotherapy do not reduce the rate of ORNJ, regardless of tooth condition, and may even increase the overall risk of ORNJ (Beacher & Sweeney, 2018; D. T. Chang et al., 2007; Sulaiman et al., 2003; Villa & Akintoye, 2018; Wahl, 2006). Above all, the healing time before the start of radiotherapy seems to be an important factor. Wound healing must be adjusted to the number of extractions, as multiple extractions should have a longer time to heal to prevent ORNJ (Koga et al., 2008). Adequate wound healing should be ensured whenever possible but not unnecessarily delay the beginning of radiotherapy (Mainali et al., 2011; Rothstein, 2005). Our protocol provides a minimum interval of 10 days between extraction and radiotherapy.

Side effects of radiotherapy are unavoidable. They depend on the radiation field and dose and may have severe negative impacts on oral quality of life (Elting et al., 2008). The most common acute complications are radiodermatitis (95.0%) (Singh et al., 2016) and stomatitis (80%–100%) (Trotti et al., 2003). A few months after treatment, xerostomia (64.0%) (Dirix et al., 2006) and dysphagia (50.0%) can occur (García‐Peris et al., 2007). Late complications evolve months to years after treatment. Most frequent to develop are ulcers (4.7%) and ORNJ of the jaw (1%−37.5%) (Schuurhuis et al., 2015; Sciubba & Goldenberg, 2006). In contrast to other reports, we observed low rates of acute, midterm, and late complications during follow‐up. In particular, osteoradionecrosis occurred in only 2.7% of patients; this value is in the lower incidence range of previous publications (Schuurhuis et al., 2015; Sciubba & Goldenberg, 2006).

Several reasons explain the low rate of ORNJ in this study. The risk of developing ORJN depends on the radiation technique and dose. A total dose of >75 Gy (J. A. Chen et al., 2016) or >70 Gy and a mean dose of >40 Gy to the mandibular bone (Gomez et al., 2011) are described as independent risk factors for ORNJ. A V60 > 14%, meaning that more than 14% of the mandible receives 60 Gy or more, has been reported to result in a higher ORNJ rate (Kubota et al., 2021). Other studies have demonstrated higher ORNJ rates with V44 ≥ 42% or V58 ≥ 25% (MD Anderson Head and Neck Cancer Symptom Working Group, 2017), confirming that tumor localization in the oropharynx and oral cavity is another risk factor; since these tumors are close to the mandibular bone, they result in higher doses to this bone (Kubota et al., 2021). However, with modern treatment planning systems and image‐guided radiotherapy using IMRT or VMAT techniques, reduction of the dose to the mandibular bone is possible and has resulted in lower ORNJ rates in recent studies (Caparrotti et al., 2017; Owosho et al., 2017). All of the patients in this study were treated with IMRT or VMAT techniques, which might explain the low rates of ORNJ in our study and emphasizes the potential importance of modern radiation planning.

Other studies have shown that the development of ORNJ usually starts 6–12 months after radiotherapy and is fully developed after 6–24 months (Nadella et al., 2015). In the present study, the mean follow‐up was 16.8 ± 10.7 months; as some cases may have developed at later time points, we may not have captured all ORNJ cases. Moreover, we had a radical approach to teeth that were not worth preserving, and the resulting high extraction rate may be responsible for the low ORNJ rate. These findings are in line with those of Ben‐David et al., who postulated that extractions of carious and nonrestorable teeth are the cornerstone of dental management before planned radiation to prevent ORNJ (Ben‐David et al., 2007).

Notably, 45%–58% of patients show signs of preradiation depression, and 7% show severe preradiation anxiety (A. M. Chen et al., 2009). In addition to suffering tumor surgery, radiation, possibly chemotherapy, and the accompanying pain, patients suffer a significant reduction in quality of life following tooth loss (Gerritsen et al., 2010). This observation implies that in the case of planned extractions, the additional psychological stress and reduction in quality of life should always be taken into account.

In summary, we observed a low rate of acute and late oral complications after radiotherapy of the head and neck region based on our standardized protocol for preradiotherapy dental management. Whether this low rate was the result of the short follow‐up period, the rather aggressive tooth extraction policy, or the modern radiation planning remains unclear. As multiple extractions are associated with a reduced quality of life, an aggressive extraction policy should be individually applied considering comorbidities and life expectancy.

## 5 STUDY LIMITATIONS

Our study was a retrospective analysis of data prospectively managed in a specific database following a standardized dental management protocol. Follow‐up was entirely based on retrospective analysis of available hospital records and was only 86.0% complete. Thus, complications, as well as death rates, may have been underestimated. Furthermore, it is not clear whether the radiation dose that the patient ultimately received is consistent with the dose initially planned by the radiation therapist. As complications may occur years after radiotherapy, our short follow‐up period may in part explain the low frequency of osteoradionecrosis. The broad variations in both the approach to dental management in patients undergoing radiotherapy of the head and neck region and the subsequent complication rates call for a randomized controlled trial comparing a conservative and more invasive dental management approach with current modern radiotherapy techniques.

## AUTHOR CONTRIBUTIONS

Lea Hoffmann wrote the manuscript and was responsible for data collection. Sebastian N. Marschner provided the current state of radiotherapy. Tamara K. Kakoschke provided the aspects of oral and maxillofacial surgery. Reinhard Hickel developed the idea and the experimental design. Hisham Sabbagh contributed substantially to the manuscript. Uta C. Wölfle was responsible for the data collection and contributed substantially to the manuscript.

## CONFLICT OF INTEREST

The authors declare no conflict of interest.

## ETHICS STATEMENT

The study was approved by the ethics committee of Ludwig Maximilian University (Ref. No 20‐778). We certify that the study was performed in accordance with the ethical standards laid down in the 1964 Declaration of Helsinki and its later amendments or comparable ethical standards.

## Data Availability

Data supporting the findings of this study are available from the corresponding author (Dr. Lea Hoffmann) upon request.
